# Proton gradient across the chloroplast thylakoid membrane governs the redox regulatory function of ATP synthase

**DOI:** 10.1016/j.jbc.2024.107659

**Published:** 2024-08-14

**Authors:** Takatoshi Sekiguchi, Keisuke Yoshida, Ken-ichi Wakabayashi, Toru Hisabori

**Affiliations:** 1 Laboratory for Chemistry and Life Science, Institute of Innovative Research, Tokyo Institute of Technology, Yokohama, Japan; 2 School of Life Science and Technology, Tokyo Institute of Technology, Yokohama, Japan; 3 International Research Frontiers Initiative, Tokyo Institute of Technology, Yokohama, Japan

**Keywords:** chloroplast ATP synthase, proton electrochemical gradient, redox regulation, thioredoxin

## Abstract

Chloroplast ATP synthase (CF_o_CF_1_) synthesizes ATP by using a proton electrochemical gradient across the thylakoid membrane, termed Δ*μ*H^+^, as an energy source. This gradient is necessary not only for ATP synthesis but also for reductive activation of CF_o_CF_1_ by thioredoxin, using reducing equivalents produced by the photosynthetic electron transport chain. Δ*μ*H^+^ comprises two thermodynamic components: pH differences across the membrane (ΔpH) and the transmembrane electrical potential (ΔΨ). In chloroplasts, the ratio of these two components in Δ*μ*H^+^ is crucial for efficient solar energy utilization. However, the specific contribution of each component to the reductive activation of CF_o_CF_1_ remains unclear. In this study, an *in vitro* assay system for evaluating thioredoxin-mediated CF_o_CF_1_ reduction is established, allowing manipulation of Δ*μ*H^+^ components in isolated thylakoid membranes using specific chemicals. Our biochemical analyses revealed that ΔpH formation is essential for thioredoxin-mediated CF_o_CF_1_ reduction on the thylakoid membrane, whereas ΔΨ formation is nonessential.

In chloroplasts, solar energy is converted to chemical energy *via* the photosynthetic electron transport chain in the thylakoid membrane. During this process, reducing equivalents are stored as NADPH, and a proton electrochemical gradient (Δ*μ*H^+^) forms across the membrane. Termed the proton-motive force, Δ*μ*H^+^ drives chloroplast F_o_F_1_-ATP synthase (CF_o_CF_1_) to catalyze ATP synthesis (1, 2). Therefore, CF_o_CF_1_ is a pivotal enzyme for energy conversion in photosynthesis. Moreover, its activity undergoes precise regulation to maintain efficient chemical energy production under varying light conditions.

Δ*μ*H^+^ comprises two thermodynamic components: pH differences across the membrane (ΔpH) and the transmembrane electrical potential (ΔΨ). Both components were reported to be kinetically equivalent in F_o_F_1_ from thermophilic bacteria (3), and early investigations into CF_o_CF_1_ revealed that both contribute to ATP synthesis by this enzyme complex (4, 5, 6). Notably, CF_o_CF_1_ exhibits thiol-based redox regulation, distinguishing it among all F_o_F_1_ enzymes across species (7, 8), with this regulation system closely linked to Δ*μ*H^+^. The central axis of CF_o_CF_1_, the γ subunit (CF_1_-γ), harbors a pair of redox-active cysteines (Cys^199^ and Cys^205^ in *Spinacia oleracea*) (9, 10). To activate CF_o_CF_1_, chloroplast thioredoxin (Trx) reduces this Cys pair, using reducing equivalents from photosynthetic electron transport reactions (7, 11). In this Trx-dependent activation process, Δ*μ*H^+^ formation is essential for CF_1_-γ reduction (12, 13, 14). Conversely, we previously revealed that Δ*μ*H^+^ dissipation stimulates CF_1_-γ oxidation (15, 16), facilitated by chloroplast oxidizing factors, such as Trx-like proteins (16, 17, 18). Consequently, CF_o_CF_1_ activity is finely tuned to activate only during photosynthetic conditions, promptly deactivating in the dark when Δ*μ*H^+^ formation ceases. However, the relationship between CF_o_CF_1_ redox regulation and the Δ*μ*H^+^ components, *i.e.*, ΔpH or ΔΨ, remains unexplored.

In chloroplasts, ΔpH plays an important role in regulating photosynthetic performance. Specific ion transporters, such as two-pore potassium channel three and voltage-dependent chloride channel 1, present in the thylakoid membrane contribute to the movement of counter ions for dissipating ΔΨ (19, 20), maintaining a high ΔpH relative to ΔΨ. Acidification of the lumen (*i.e.*, ΔpH formation) regulates the electron transfer activity of the cytochrome *b*_6_*f* complex (21, 22). Additionally, ΔpH serves as a key signal for initiating nonphotochemical quenching (NPQ), which functions as a photoprotection mechanism, especially the qE component (23). Therefore, green plants must maintain a balanced ΔpH-to-ΔΨ ratio within Δ*μ*H^+^ for safe light utilization.

This study focuses on the thermodynamic components of Δ*μ*H^+^ required for CF_1_-γ reduction by Trx. Our investigation involved manipulating Δ*μ*H^+^ bias in isolated spinach thylakoids using specific chemicals. We also established an *in vitro* assay system using freshly prepared thylakoid membranes to facilitate CF_1_-γ reduction by Trx on the membrane.

## Results and discussion

### Establishing biased ΔμH^+^ conditions in the thylakoid membrane using ionophores

**Figure 1:**
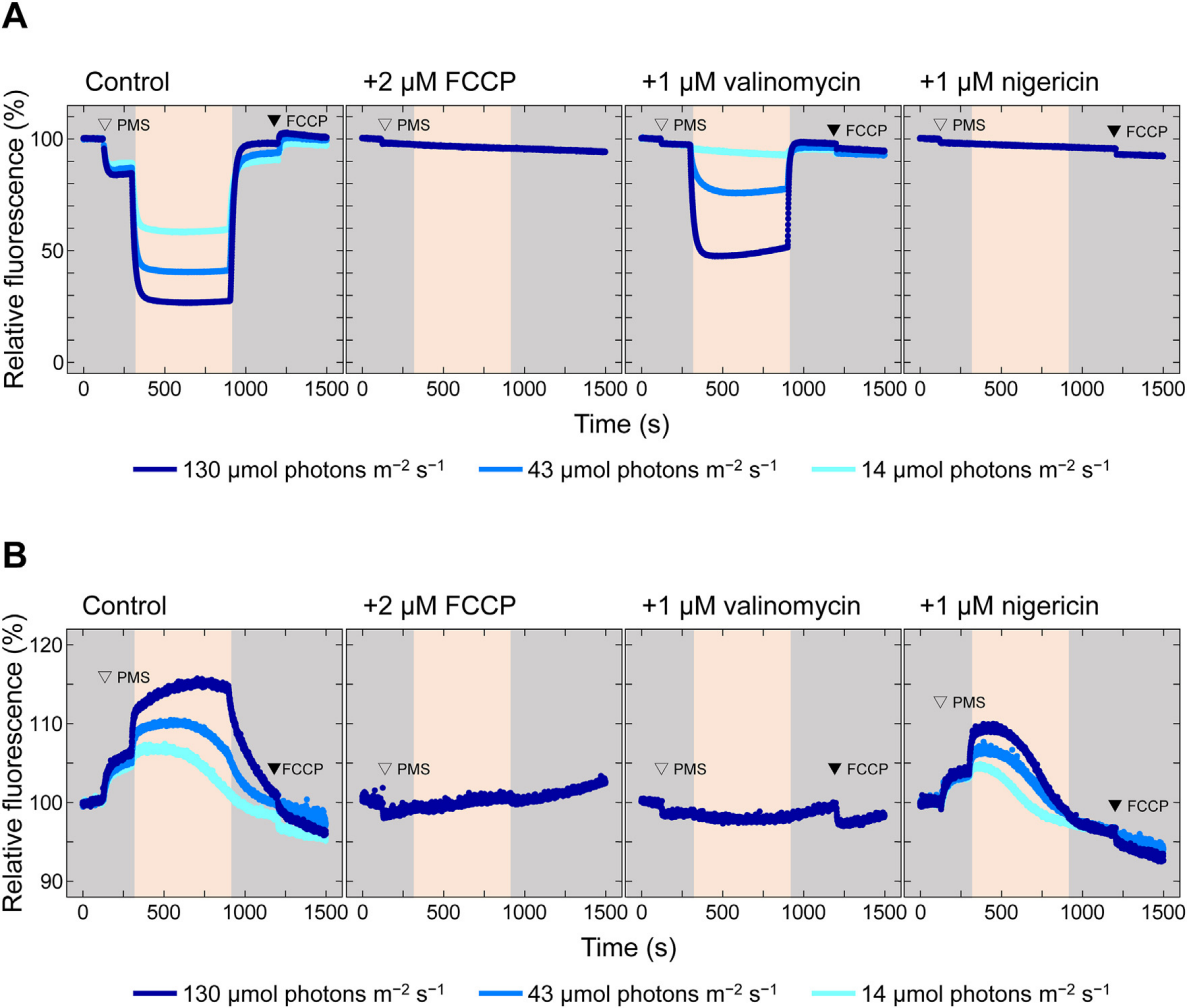
**Ionophore effects on light-induced Δ*μ*H**^**+**^**in the thylakoid membrane**. Observations of ΔpH and ΔΨ across the thylakoid membrane induced by *red light* irradiation. (*A*) ΔpH measurement using ACMA. Thylakoid membranes (5 μg chlorophyll (Chl)/ml) were incubated with 0.3 μg/ml ACMA, and ACMA fluorescence intensity (λ_ex_ = 410 nm, λ_ex_ = 480 nm) was monitored. (*B*) ΔΨ measurement using ANS. Thylakoid membranes (5 μg Chl/ml) were incubated with 100 μM ANS, and ANS fluorescence intensity (λ_ex_ = 330 nm, λ_ex_ = 455 nm) was monitored. *Gray* sections indicate *dark* conditions, whereas *highlighted* sections indicate *red light* irradiation periods. *Open* and *closed triangles* represent the addition of 2 μM 1-methoxy PMS and 1 μM FCCP, respectively.

H^+^ translocation across the membrane concurrently generates ΔpH and ΔΨ. Through the application of ionophores, such as nigericin, valinomycin, or the uncoupler FCCP, we can establish conditions where either ΔpH or ΔΨ forms in the thylakoid membrane or neither form. Using this approach, we aimed to discern differences in the contributions of ΔpH and ΔΨ to CF_1_-γ redox regulation. Specifically, we used the fluorescent reagents 9-amino-6-chloro-2-methoxyacridine (ACMA) and 8-anilinonaphthalene-1-sulfonic acid (ANS) to monitor ΔpH and ΔΨ formation on the thylakoid membrane, respectively (Fig. 1). ACMA fluorescence intensity decreases upon protonation (24, 25), whereas ANS fluorescence increases upon potentiated membrane binding (26, 27). The addition of the artificial electron mediator 1-methoxy-5-methylphenazinium methylsulfate (PMS) to the thylakoid membrane induces Δ*μ*H^+^ formation across the membrane under light due to pseudocyclic electron transport around photosystem I (28). We then monitored ΔpH formation by observing the ACMA fluorescence decrease (120 s; Fig. 1*A*, Control). Subsequently, red light irradiation further enhanced ΔpH formation (300 s; highlighted area of the graph in Fig. 1), dependent on light intensity (14–130 μmol photons m^−2^ s^−1^). ΔpH remained constant during red light irradiation and dissipated upon its cessation (900 s), finally returning to initial levels upon FCCP addition (1200 s). Similar temporal events were observed for ΔΨ formation using ANS (Fig. 1*B*, Control), with ΔΨ gradually declining under low light conditions (14 and 43 μmol photons m^−2^ s^−1^).

The effects of FCCP, valinomycin, and nigericin on ΔpH and ΔΨ formation under the aforementioned light conditions were then examined. FCCP selectively permeates H^+^ across the membrane, abolishing both ΔpH and ΔΨ (*i.e.*, Δ*μ*H^+^). Pretreatment of the thylakoid membrane with FCCP almost abolished fluorescence changes in both ACMA and ANS (Fig. 1, *A* and *B*, +2 μM FCCP). Nigericin disrupts only ΔpH formation by exchanging lumen H^+^ for stromal K^+^, whereas valinomycin disrupts only ΔΨ formation by selectively transporting K^+^. To use these ionophores, 50 mM KCl was supplemented in the grinding buffer for thylakoid membrane preparation. Pretreatment of the thylakoid membrane with valinomycin led to ACMA but not ANS fluorescence changes in a light-dependent manner (Fig. 1, *A* and *B*, +1 μM valinomycin). Conversely, pretreatment of the thylakoid membrane with nigericin caused minimal ACMA fluorescence change (Fig. 1*A*, +1 μM nigericin). In contrast, changes in ANS fluorescence resembled those of control conditions under any examined light conditions, albeit to a lesser extent (Fig. 1*B*, +1 μM nigericin). These results highlight the successful identification of varying ionophore actions in the thylakoid membrane as well as the conditions distinguishing both ΔpH and ΔΨ.

Historically, the electrochromic shift in endogenous carotenoid absorbance at 515 nm (Δ*A*_515_) has been used to monitor the transmembrane ΔΨ level (29). Δ*A*_515_ can be observed by briefly exposing isolated thylakoids to actinic light flashes. However, we employed ACMA and ANS in this study. This is because these fluorescent reagents have a history of being used in combination with ionophores and uncoupler to measure the enzymatic activity of ATP synthase and other ion transporters in *in vitro* studies, and their effectiveness has been confirmed (16, 30, 31, 32).

### Trx-mediated CF_1_-γ reduction on the thylakoid membrane fails to occur without *Δ*pH formation

**Figure 2:**
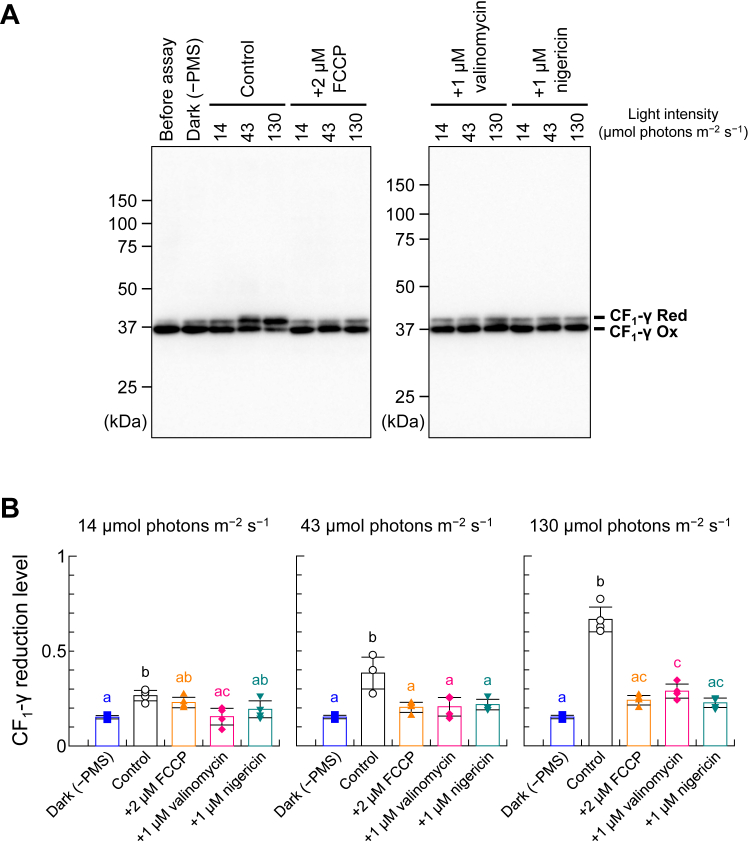
**Characterization of Δ*μ*H**^**+**^**-dependent thylakoid CF**_**1**_**-γ reduction by Trx.***A*, determination of the thylakoid CF_1_-γ redox state. CF_1_-γ in the thylakoid membrane (5 μg Chl/ml) was reduced by 1 μM Trx-*f* and 100 μM DTT in the presence of each ionophore under the indicated *light* conditions for 5 min. After free thiol modification with AMS, proteins underwent nonreducing sodium dodecyl sulfate–polyacrylamide gel electrophoresis (SDS-PAGE) followed by western blotting with anti-CF_1_-γ antibodies. *Ox*, oxidized form; *Red*, reduced form. *B*, quantification of CF_1_-γ reduction levels for the data shown in (*A*). Data represent means ± standard deviations (SDs; n = 3–4). Different letters indicate significant differences (*p* < 0.05; one-way analysis of variance and Tukey’s honest significance differences test).

Using the abovementioned conditions to distinguish ΔpH and ΔΨ (Fig. 1), we performed Trx-mediated reduction assays of CF_1_-γ on the thylakoid membrane (Fig. 2). *In vitro* reduction experiments involving CF_1_-γ were performed using the same concentrations of thylakoid membrane [5 μg chlorophyll (Chl)/ml] and 1-methoxy PMS (2 μM) shown in Figure 1. The redox state of CF_1_-γ was determined using 4-acetamido-4′-maleimidylstilbene-2,2′-disulfonic acid (AMS), as described previously (16). CF_1_-γ is known to be reduced by Trx-*f*, a major isoform of chloroplast Trx proteins (33, 34). This reduction process requires prior Δ*μ*H^+^ formation across the thylakoid membrane (7, 13, 14). As expected, CF_1_-γ on the thylakoid membrane was not reduced by 1 μM Trx-*f* in the presence of 100 μM dithiothreitol (DTT) under dark conditions without PMS [Fig. 2*B*, Dark (−PMS)]. Upon performing the reduction assay under the same light conditions shown in Figure 1 (14–130 μmol photons m^−2^ s^−1^), we observed a light intensity-dependent reduction of CF_1_-γ (Fig. 2*B*, Control). Thus, the CF_1_-γ reduction level was controlled by the extent of Δ*μ*H^+^ across the thylakoid membrane. Next, we investigated the effects of an uncoupler and ionophores under the conditions shown in Figure 1 (2 μM FCCP, 1 μM valinomycin, or 1 μM nigericin), and found that CF_1_-γ reduction by Trx was significantly inhibited by these chemicals under all tested light conditions.

**Figure 3:**
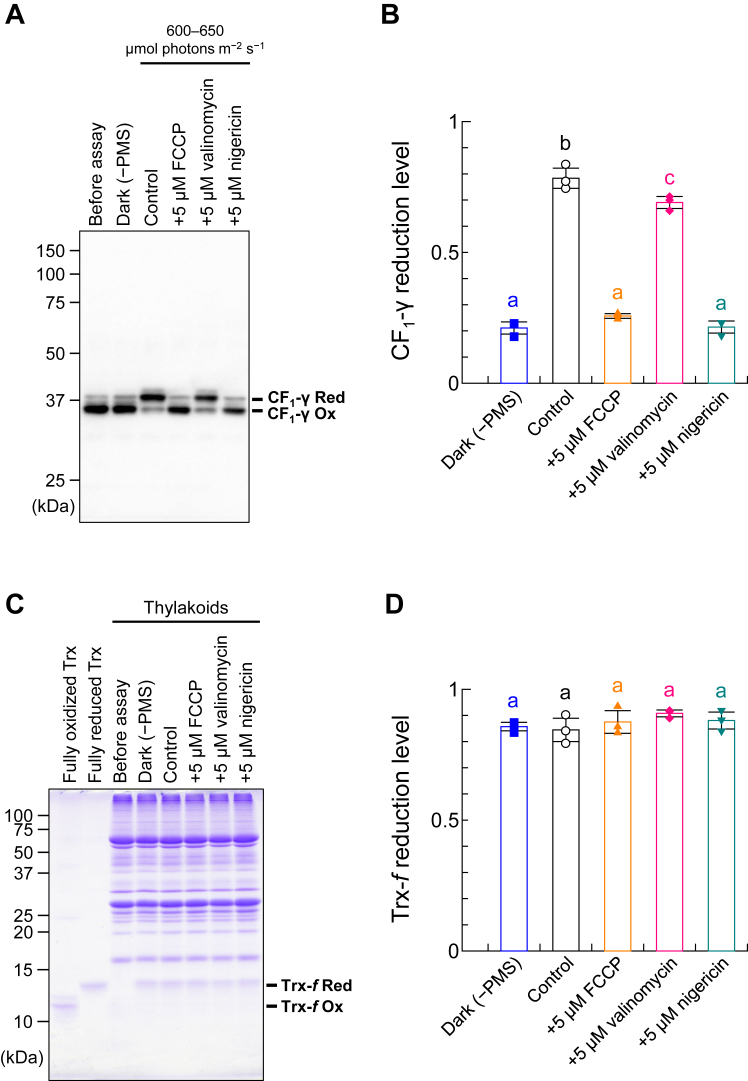
***In vitro* CF_1_-γ reduction using high-concentration thylakoid membranes**. *A* and *C*, determination of the thylakoid CF_1_-γ and Trx-*f* redox states. CF_1_-γ in the thylakoid membrane (50 μg Chl/ml) was reduced by 1 μM Trx-*f* and 100 μM DTT in the presence of each ionophore under 600 to 650 μmol photons m^−2^ s^−1^ for 5 min. After free thiol modification with AMS, proteins underwent nonreducing SDS-PAGE, and the redox state was visualized *via* western blotting with anti-CF_1_-γ antibodies (*A*) or Coomassie Brilliant Blue staining (*C*). *Ox*, oxidized form; *Red*, reduced form. *B* and *D*, quantification of the CF_1_-γ and Trx-*f* reduction levels for the data shown in (*A*) and (*C*), respectively. Data represent means ± SDs (n = 3). Different letters indicate significant differences (*p* < 0.05; one-way ANOVA and Tukey’s HSD test).

**Figure 4:**
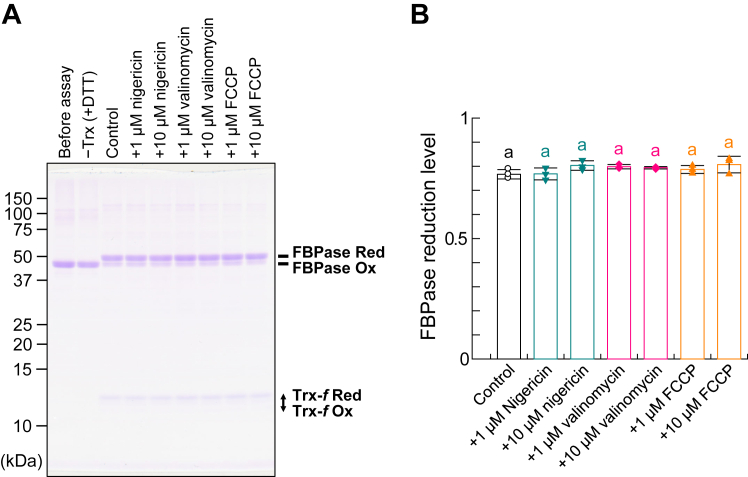
**FBPase reduction by Trx-*f* in the presence of ionophores**. *A*, FBPase (2 μM) was incubated with 1 μM Trx-*f* and 100 μM DTT for 30 min. After free thiol modification with AMS, proteins underwent nonreducing SDS-PAGE followed by Coomassie Brilliant Blue staining. *B*, quantification of the FBPase redox state for the data shown in (*A*). Data represent means ± SDs (n = 3). Different letters indicate significant differences (*p* < 0.05; one-way ANOVA and Tukey’s HSD test).

To further explore the relationship between Δ*μ*H^+^ and CF_1_-γ reduction, we used higher concentrations of the thylakoid membrane (50 μg Chl/ml) and 1-methoxy PMS (100 μM) under more intense light conditions (600–650 μmol photons m^−2^ s^−1^), following our previous study (16, 34) (Fig. 3). Although CF_1_-γ was not reduced in the dark [Fig. 3, *A* and *B*, Dark (−PMS)], it was reduced by approximately 80% when 1 μM Trx and 100 μM DTT were added in the light (Fig. 3, *A* and *B*, Control). When FCCP or nigericin was added to the thylakoid membrane beforehand, CF_1_-γ was not reduced, similar to dark conditions (Fig. 3, *A* and *B*, +5 μM FCCP, +5 μM nigericin) as well as the results shown in Figure 2. Both FCCP and nigericin dissipated ΔpH formation across the thylakoid membrane, as confirmed *via* fluorescence measurements (Fig. 1*A*, +2 μM FCCP, +1 μM nigericin). However, even in the presence of valinomycin, CF_1_-γ was reduced by approximately 70% (Fig. 3, *A* and *B*, +5 μM valinomycin). Under these experimental conditions, Trx-*f* was almost completely reduced when sufficient amounts of DTT were added (Fig. 3, *C* and *D*). We also examined whether FCCP and ionophores affect the Trx-dependent reduction of other target enzymes by testing FBPase reduction *via* Trx-*f* in the presence of FCCP or ionophores. Notably, FBPase is the major target enzyme of Trx-*f* in chloroplasts (35, 36). As shown in Figure 4, Trx-*f* reduced FBPase efficiently even in the presence of FCCP or ionophores in the reaction mixture, implying that the inhibited CF_1_-γ reduction shown in Figures 2 and 3 was due to ΔpH dissipation caused by FCCP or nigericin. Hence, ΔpH but not ΔΨ formation across the thylakoid membrane was required for CF_1_-γ reduction. The varying results for valinomycin with different thylakoid membrane concentrations may be attributed to differences in membrane stability under the respective experimental conditions. Higher membrane concentrations may maintain stability and reduce H^+^ leakage.

**Figure 5:**
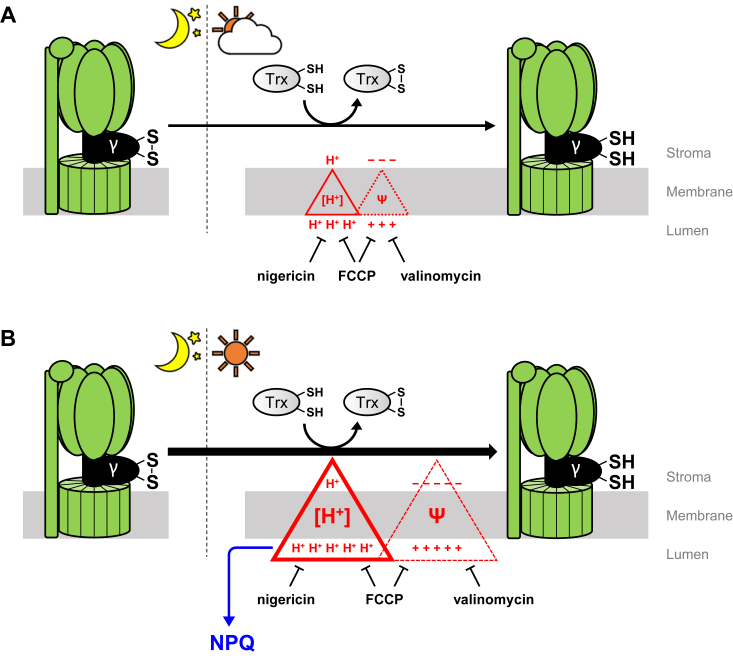
**An overview of the relationship between the redox regulation of CF**_**1**_**-γ and ΔpH formation.** The formation of Δ*μ*H^+^ when CF_1_-γ is reduced by Trx under *low light* (*A*) or *high light* (*B*) conditions are illustrated. *Triangles* represent the extent of ΔpH across the thylakoid membrane, and *dashed triangles* represent the extent of ΔΨ across the thylakoid membrane.

Our results raise an important question: does ΔΨ have any effect on CF_1_-γ reduction? As shown in Figure 2, CF_1_-γ reduction did not occur with pretreatment of either nigericin or valinomycin but was observed in the control experiment, especially under 130 μmol photons m^−2^ s^−1^. These results imply that ΔΨ supports CF_1_-γ reduction when ΔpH is low. Notably, the extent of ΔpH with added valinomycin was slightly lower than that under control conditions (Fig. 1*A*, Control, +1 μM valinomycin). However, CF_1_-γ was reduced when valinomycin was added under more intense light conditions (Fig. 3, *A* and *B*, +5 μM valinomycin). Thus, when sufficient ΔpH forms across the thylakoid membrane, CF_1_-γ reduction occurs regardless of ΔΨ formation, *i.e.*, the extent of ΔpH governs the CF_1_-γ reduction process. This reduction process observed in this study under different light conditions was illustrated, along with the effect of ionophores and uncoupler used (Fig. 5). Alkaline conditions near the surface of the thylakoid membrane may be favorable for the dithiol-disulfide exchange reaction between CF_1_-γ and Trx. However, this detailed molecular mechanism is not yet clear, and further studies are required. As ΔpH across the thylakoid membrane induces NPQ, it is considered crucial in plant physiology, whereas ΔΨ is used exclusively to regulate ΔpH. Overall, the ability to activate CF_o_CF_1_ reductively *via* Trx under fluctuating light conditions without relying on ΔΨ formation is likely advantageous for plants.

## Experimental procedures

### Preparation of thylakoid membranes from spinach leaves

Thylakoid membranes were prepared from spinach (*S. oleracea*) as previously described (34) but with slight modifications. Fresh market spinach was washed thoroughly and left overnight in the dark at 4 °C. Harvested leaves (approximately 10 g fresh weight) were homogenized three times for 3 s in a mixer with 200 ml of grinding buffer [50 mM Tricine-NaOH (pH 7.5), 0.4 M sucrose, 5 mM MgCl_2_, 10 mM NaCl, and 50 mM KCl]. The homogenate was filtered through four layers of gauze and centrifuged at 3,000*g* and 4 °C for 10 min. The pellet was then resuspended in the grinding buffer and centrifuged at 300*g* and 4 °C for 1 min, after which the supernatant was collected and centrifuged at 3,000*g* and 4 °C for 10 min. After the abovementioned washing step was repeated once, the resulting pellet was resuspended in the grinding buffer to achieve a Chl concentration of 0.5 mg/ml. The preparation was kept in the dark on ice for at least 1 h before the assay.

### Monitoring the formation of proton gradients and membrane potential gradients across thylakoid membranes

We measured ΔpH and ΔΨ across the thylakoid membrane using ACMA and ANS, respectively. The grinding buffer for thylakoid preparation was used as the reaction mixture, with the reaction performed at 25 °C. Red light irradiation at 660 nm induced the formation of these thermodynamic gradients across the thylakoid membrane. Before use, ACMA was solubilized at 30 μg/ml in 100% ethanol and stored at −80 °C. ANS was prepared as a 10 mM solution in 10% DMSO and stored at room temperature. The emitted fluorescence of ACMA (λ_ex_ = 410 nm, λ_ex_ = 480 nm) and ANS (λ_ex_ = 330 nm, λ_ex_ = 455 nm) was measured using a FP-8500 spectrofluorometer (Jasco).

Stored ACMA or ANS solution (20 μl) and 50 μl of 200 μg Chl/ml thylakoid membrane were added to 1910 μl of the grinding buffer in a grass cuvette and left to stand for stabilization of the fluorescence signal. At 120 s after initiating the measurement, 20 μl of 200 μM 1-methoxy PMS was added to the mixture. The final concentrations in the cuvette were 5 μg Chl/ml of thylakoid membranes, 2 μM 1-methoxy PMS, and 0.3 μg/ml ACMA (in 0.1% ethanol) or 100 μM ANS (in 0.01% DMSO). Subsequently, the reaction mixture was irradiated with red light from a direction perpendicular to the cuvette using a light-emitting diode (LED) at 300 s and terminated at 900 s. The photon flux density of the red light is shown in Figure 1. At 1200 s, 2 μl of 2 mM FCCP was added to the mixture to confirm whether Δ*μ*H^+^ was dissipated compared with the initial condition. The initial fluorescence intensity of each trace was normalized to 100% using the average of the data from approximately 30 to 90 s, *i.e.*, when fluorescence intensity was relatively stable.

### Recombinant protein preparation

The recombinant proteins used in this study, spinach Trx-*f* and *Arabidopsis thaliana* FBPase, were prepared as described previously (34, 37). Protein concentrations were determined using a BCA protein assay (Pierce).

### *In vitro* assay of Trx-mediated CF_o_CF_1_ reduction

For the reduction assay, the grinding buffer for thylakoid membrane preparation was used as the reaction mixture, and the reaction was performed at 25 °C. Prior to the assay, 10 μM Trx-*f* was incubated with 1 mM DTT in the grinding buffer for 5 min. Subsequently, 200 μl of the Trx-*f*/DTT mixture and 50 μl of 200 mg Chl/ml thylakoid membrane were added to 1730 μl of the grinding buffer and incubated for 1 min. Next, 20 μl of 200 μM 1-methoxy PMS was added to the mixture. The final concentrations in the mixture were 5 μg Chl/ml thylakoid membranes, 2 μM 1-methoxy PMS, 100 μM DTT, and 1 μM Trx-*f*. This mixture was irradiated with red light at 660 nm for 5 min using an LED to initiate Δ*μ*H^+^ formation across the thylakoid membrane. The photon flux density of the red light is shown in Figure 2. Similar experiments were performed using higher thylakoid membrane concentrations (final concentration, 50 μg Chl/ml) and 1-methoxy PMS (final concentration, 100 μM) under more intense light conditions (600–650 μmol photons m^−2^ s^−1^). Following the *in vitro* assay, proteins were precipitated using 10% (w/v) trichloroacetic acid to stop the reduction reaction.

### *In vitro* assay of Trx-mediated FBPase reduction

For FBPase reduction, a medium containing 50 mM Tris-HCl (pH 7.5) and 50 mM NaCl was used, with the reaction performed at 25 °C. Protein and reducing agent concentrations as well as reaction times are described in the Figure 4 legend.

### Determination of the protein redox state

The protein redox state was determined by labeling free thiols with AMS and employing sodium dodecyl sulfate–polyacrylamide gel electrophoresis (for Trx-*f* and FBPase) or immunoblotting (for CF_1_-γ), as described previously (34, 37). The antibody against CF_1_-γ were prepared using recombinant *Arabidopsis* CF_1_-γ (His-tagged at the C terminus) as an antigen, and its specificity is indicated in our former paper (38). Chemiluminescence was detected using horseradish peroxidase–conjugated secondary antibodies and ECL Prime (Cytiva) and visualized on a LAS 3000 Mini Imaging System (Fuji Film). The resultant band intensities were quantified using ImageJ. The reduction level was calculated as the ratio of the reduced form to the total form. The data in Figure 2, Figure 3, Figure 4 were statistically analyzed using one-way analysis of variance and Tukey’s honest significance differences test (*p* < 0.05). Statistical analyses were performed using an online calculator at iCalcu.com (https://www.icalcu.com/stat/anova-tukey-hsd-calculator.html).

## Data availability

All data are contained within the article.

## Conflict of interest

The authors declare that they have no conflicts of interest regarding the content of this article.
